# Species diversity of mosquitoes (*Diptera*: *Culicidae*), larval habitat characteristics, and potential as vectors for lymphatic filariasis in Central Bengkulu Regency, Indonesia

**DOI:** 10.14202/vetworld.2024.2115-2123

**Published:** 2024-09-20

**Authors:** Deri Kermelita, Upik Kesumawati Hadi, Susi Soviana, Risa Tiuria, Supriyono Supriyono

**Affiliations:** 1 Parasitology and Medical Entomology Laboratory, Animal Biomedicine Study Program, Graduate School, IPB University, Bogor, Indonesia; 2 Department of Animal Infectious Diseases and Veterinary Public Health, Faculty of Veterinary Medicine, IPB University, Bogor, Indonesia

**Keywords:** Bengkulu, characteristics, diversity, filariasis, habitat, vector

## Abstract

**Background and Aim:**

Lymphatic filariasis (LF) is a vector-borne disease in various regions of Indonesia. The transmission dynamics within a locality are intricately linked to the presence of the pathogen (microfilaria), definitive host (humans), intermediate host (mosquitoes), reservoir, and environmental factors. The geographic landscape of Central Bengkulu Regency, which is characterized by plantations, marshlands, and forests, serves as a suitable habitat for mosquitoes. Understanding species diversity, vector behaviors, habitat characteristics, and microfilarial presence is crucial for devising effective and efficient control strategies. This study aimed to identify species diversity, assess biting patterns, characterize larval habitats, and detect microfilarial presence in mosquitoes.

**Materials and Methods:**

Mosquito collection was conducted using human landing collection (HLC) and resting collection indoors and outdoors for 6 months at a frequency of twice monthly from November 2022 to May 2023. The larvae were collected using dippers and pipettes. Adult mosquitoes and larvae were identified at the species level and analyzed using diversity indices. The measured larval bioecological parameters included physical, chemical, and biological conditions. The mosquito density obtained through HLC was calculated using the man-hour density (MHD) and man-biting rate (MBR) formulas. The presence of microfilaria was confirmed using a polymerase chain reaction.

**Results:**

A total of 808 adult mosquitoes from five genera and 18 species were captured, along with 485 larvae from four genera and eight species. The mosquito diversity was moderate. The dominant adult species included *Armigeres subalbatus* (44.8%), whereas *Aedes albopictus* (25.4%) and *Ar. subalbatus* (22.3%) were abundant larvae. The highest larval density was observed in natural ponds. The average MBR was three mosquitoes per person per night, with fluctuating nightly activity (mean MHD of 1.8 mosquitoes per person per hour). Larval habitats had temperatures of 25.4°C–28.7°C, illumination of 224–674 lx, and pH of 7.1–7.9, with over half being turbid and nearly two-thirds lacking predators. Microfilariae were not detected in the tested mosquitoes.

**Conclusion:**

The presence of mosquitoes, their habitat, and the high density of *Ar. subalbatus* contributes to the transmission of LF in Central Bengkulu Regency, Indonesia.

## Introduction

Lymphatic filariasis (LF) is a chronic infectious disease caused by infection of the microfilariae *Wuchereria bancrofti*, *Brugia malayi*, and *Brugia timori* by mosquitoes of the genera *Mansonia*, *Aedes*, *Culex*, *Anopheles*, and *Armigeres*. The World Health Organization classifies LF as a neglected tropical disease that remains a public health issue worldwide [1]. An estimated 120 million individuals across 83 countries globally, particularly those in tropical and subtropical regions, have been infected by filarial nematodes, with 60% of the reported cases concentrated in Southeast Asia. Approximately 1.3 billion people worldwide remain vulnerable to filariasis, resulting in disability in 40 million individuals [1]. Filariasis is prevalent in all provinces of Indonesia, with 9906 recorded cases in 2020 dispersed across 34 provinces. The highest incidence of filariasis occurs in the eastern part of Indonesia, notably in Papua (3615 cases) and East Nusa Tenggara (1534 cases). Conversely, in the western region of Indonesia, West Java exhibits the highest number of filariasis cases at 641. Provinces with fewer than five recorded cases of filariasis include Bali, the Special Region of Yogyakarta, North Kalimantan, and Gorontalo [2].

From an epidemiological perspective, the spread of filariasis in an area is influenced by several factors: the presence of the microfilarial agent, humans as definitive hosts, mosquitoes as intermediate hosts, the existence of reservoirs, and conducive environmental conditions [3]. The diversity of mosquito species, their density, biting rates, and ability to acquire and transmit microfilariae, which develop into the infective stage (L3), significantly affect the spread of filariasis. Filariasis is more common in areas with poor sanitation [3, 4]. Efforts must focus not only on mass drug administration and integrated vector control to break the transmission chain. One challenge in Indonesia is its diverse geography and demographics, which lead to variations in vector species across regions [5]. Bengkulu Province is an endemic region of filariasis in Indonesia, with 66 reported cases in 2020 [6]. Central Bengkulu Regency is a subdivision within the province that is categorized as non-endemic for filariasis. However, in 2020, seven filariasis cases were reported in Central Bengkulu Regency, which was distributed across three districts: Karang Tinggi, Pondok Kelapa, and Pematang Tiga [6, 7]. Central Bengkulu Regency is characterized by hilly topography, with elevations reaching up to 541 m above sea level. Geographically, it is surrounded by plantations, marshlands, and forests, which provide ideal conditions for the life cycle and development of filariasis vector mosquitoes. Climatically, the regency is classified as type A (wet tropics), with humidity levels ranging from 70% to 87%. It undergoes 10 wet months annually, from October to July, with average temperatures ranging from 25°C to 27°C. Monthly rainfall varies from 230 to 620 mm and is accompanied by 10–23 rainy days per month [7].

The most effective strategy for eliminating LF cases or disrupting the transmission chain of filariasis involves comprehensive case management (mass drug administration), regular surveillance to inform vector mosquito control efforts, and environmental stewardship to eliminate mosquito breeding sites [4, 5]. Essential information required for filariasis vector control encompasses understanding mosquito species diversity and abundance, larval breeding habitats, and the prevalence of microfilariae within vectors in the study area.

Thus, this study was undertaken to delineate species diversity, assess biting behavior, characterize larval habitats, and determine the presence of microfilariae within vectors in Central Bengkulu Regency, Bengkulu Province.

## Materials and Methods

### Ethical approval

This study was approved by Ethics Committee of the Bengkulu Polytechnic of Ministry Health (KEPK/395/08/2022).

### Study period and location

**Figure-1:**
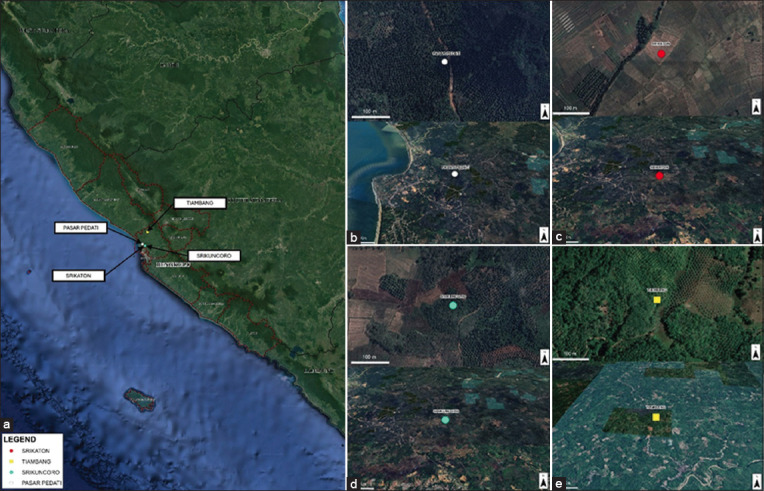
Map of Central Bengkulu Regency, Indonesia (a), shows the mosquito sampling sites: (b) Pasar Pedati village, (c) Srikaton village, (d) Srikuncoro village, and (e) Tiambang village [Source: The map was drawn using ArcGIS version 10.8, based on the Indonesia Earth Map Scale of 1:50,000].

This study was conducted from November 2022 to May 2023 in Central Bengkulu Regency (Figure-1), which comprises four villages: Srikuncoro Village (3°43'22"S, 102°17'50"E), Srikaton Village (3°43'24"S, 102°17'27"E), Pasar Pedati Village (3°42'18"S, 102°16'11"E), and Tiambang Village (3°34'19"S, 102°19'30"E).

### Mosquito collection and identification

Mosquito collection was conducted using the human landing collection (HLC) and resting collection methods for 6 months at a frequency of twice per month. HLC was conducted indoors and outdoors at night. The mosquito collectors consisted of two individuals who caught mosquitoes for 50 min every 60 min for 12 h. The collectors exposed their calves to the knees as bait for mosquitoes and then collected the resting mosquitoes using an aspirator. Mosquitoes were caught every hour and placed in labeled plastic cups. The live mosquitoes were killed using chloroform and were identified based on the morphological keys of *Aedes*, *Anopheles*, *Armigeres*, *Culex*, and *Mansonia* [8–12].

### Larval collection, habitat characteristics, parameter measurements, and species identification

Larvae were collected at potential breeding sites using 350 mL dipper and pipette. The dipper larvae were collected from more significant breeding sites with ample water. A pipette was used to collect larvae from smaller breeding sites with limited water availability. The larvae were kept in mosquito cages until they became adult mosquitoes. The larvae were identified using key mosquito morphological species in Indonesia [8–12]. The larval bioecology was observed by measuring the physicochemical and biological characteristics of various natural and artificial habitats. Physicochemical characteristics include water temperature, illumination, pH, and salinity, whereas biological characteristics encompass water conditions, vegetation, and predators [13].

### Filarial detection from mosquito samples

Mosquitoes were pooled based on species. Genomic DNA from the pool of mosquitoes was extracted using a DNA extraction kit GENEAID® Genomic DNA mini kit (Tissue) (Geneaid Biotech Ltd., Taiwan) in accordance with the manufacturer’s instructions and stored at −20°C until use. Detection of microfilariae was performed using a universal primer for the filarial worm, the internal transcribed spacer (ITS-1 Forward 5'-GGTGAACCTGCGGAAGGATC-3' and ITS-1 Reverse 5'-GCGAATTGCAGACGCATTGAG-3'), and a specific primer for *B. malayi* (Hha1 Forward 5'-GCGCATAAATTCATCAGC-3' and Hha1 Reverse 5-GCG CAAAACTTAATTACAAAAGC-3'). Amplifications were performed in 25-μL aliquots. Each polymerase chain reaction (PCR) master mix consisted of the following per reaction tube: 12.5-μL PCR Master Mix (Bioline®, United Kingdom), 1 μL of each primer pair (20 pmol/μL, Integrated DNA Technologies®, United States of America), 7.5-μL diethyl pyrocarbonate (DEPC)-treated water, and 3-μL DNA template.

The thermal cycler machine was set as follows: Pre-denaturation (94°C for 3 min), 35 cycles of denaturation (94°C for 30 s), annealing (58°C for 30 s/51°C for 30 s), elongation (72°C for 30 min), and post-elongation (72°C for 7 min).

PCR products were separated through electrophoresis on 1% agarose gels and visualized using an ultraviolet transilluminator. The mosquito samples were found to be positive for filarial nematodes and *B. malayi* microfilariae if a 482- or 322-bp DNA fragment was amplified, respectively [14].

### Statistical analysis

#### Mosquito diversity indices

The diversity of mosquitoes and larvae was calculated using various ecological indices, including the dominance index (D), Simpson’s diversity index (1-D), Shannon’s diversity index (H), and Shannon–Wiener evenness index (E). These indices were further analyzed using the PAST 4.0 Software (https://past.en.lo4d.com/download) to assess biodiversity and distribution patterns in the study area. The diversity index values were categorized into three groups: H' <1 = low species diversity, 1< H' <3 = moderate species diversity, and H' >3 = high species diversity.

#### Density and biting activity

The mosquitoes collected through HLC were quantified using the man-hour density (MHD) and man-biting rate (MBR) formulas. Descriptive analysis was conducted on the MHD and MBR values to identify the anthropophilic species of mosquitoes and their density and blood-feeding activity. The MBR and MHD were calculated as follows:


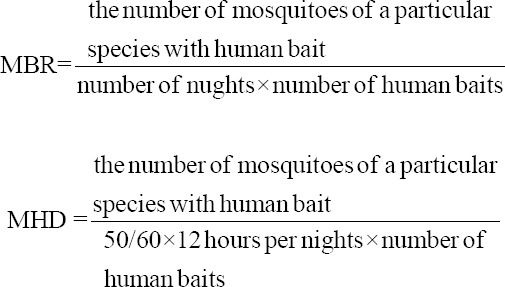


## Results

### Diversity of mosquitoes

Throughout the 6-month study period, 808 adult mosquitoes were captured, representing 18 species across five genera: *Aedes*, *Anopheles*, *Armigeres*, *Culex*, and *Mansonia*. Table-1 presents the species diversity based on collection method and location, whereas Table-2 provides comprehensive insights into the diversity indices, abundance, richness, evenness, and dominance of the captured mosquitoes.

**Table-1:** Percentage of mosquito species caught using human landing and resting collection methods in Central Bengkulu Regency, Indonesia, from November 2022 to May 2023.

Species	Number of species (%)	Human landing collection		Resting collection	
	
		Indoor (%)	Outdoor (%)	Indoor (%)	Outdoor (%)
*Aedes aegypti*	8 (1.0)	1 (1.0)	1 (0.4)	0 (0.0)	6 (1.9)
*Armigeres subalbatus*	362 (44.8)	16 (15.2)	132 (49.1)	38 (33.9)	176 (54.7)
*Culex fuscocephalus*	8 (1.0	2 (1.9)	3 (1.1)	2 (1.8)	1 (0.3)
*Culex quinquefasciatus*	327 (40.5)	53 (50.5)	95 (35.3)	56 (50.0)	123 (38.2)
*Culex hutchinsoni*	5 (0.6)	1 (1.0)	2 (0.7)	2 (1.8)	0 (0)
*Culex vishnui*	4 (0.5)	2 (1.9)	1 (0.4)	0 (0)	1 (0.3)
*Culex tritaeniorhynchus*	1 (0.1)	1 (1.0)	0 (0)	0 (0)	0 (0)
*Culex pseudovishnui*	52 (6.4)	14 (13.3)	23 (8.6)	9 (8.0)	6 (1.9)
*Culex pseudosinensis*	2 (0.2)	0 (0)	1 (0.4)	0 (0)	1 (0.3)
*Anopheles subpictus*	4 (0.5)	1 (1.0)	1 (0.4)	0 (0)	2 (0.6)
*Anopheles minimus*	3 (0.4)	1 (1.0)	0 (0)	1 (0.9)	1 (0.3)
*Anopheles vagus*	1 (0.1)	0 (0)	1 (0.4)	0 (0)	0 (0)
*Anopheles leucosphyrus*	1 (0.1)	0 (0)	1 (0.4)	0 (0)	0 (0)
*Anopheles indefinitus*	3 (0.4)	2 (1.8)	1 (0.4)	0 (0)	0 (0)
*Anopheles barbirostris*	1 (0.1)	0 (0)	1 (0.4)	0 (0)	0 (0)
*Anopheles aconitus*	1 (0.1	1 (1.0)	0 (0	0 (0)	0 (0)
*Mansonia annulata*	16 (2.0)	5 (4.8)	4 (1.5)	3 (2.7)	4 (1.2)
*Mansonia uniformis*	9 (1.1)	5 (4.8)	2 (0.7)	1 (0.9)	1 (0.3)
Total	808 (100)	105 (13)	269 (33)	112 (14)	322 (40)

Table-1 illustrates the diversity of mosquito species captured, comprising five genera and 18 species. Among these species, *Armigeres* subalbatus was predominant (44.8%), followed by *Culex* quinquefasciatus (40.5%), *Culex* pseudovishnui (6.4%), *Mansonia* annulata (2.0%), and Mansonia uniformis (1.1%). Overall, Ar. subalbatus abundance was notably higher outdoors, as captured using HLC (49.1%) and resting collection (54.7%), whereas *Cx. quinquefasciatus* abundance was significantly higher indoors using both HLC (50.5%) and resting collection (50.0%).

Table-2 shows moderate mosquito diversity in central Bengkulu, as indicated by the Shannon diversity index (H). The indoor HLC method exhibited higher Simpson’s diversity index values (0.698) than the other methods. The Shannon–Wiener evenness index (E) was 0.384, 0.240, 0.432, and 0.253. The evenness values of the mosquitoes based on the Shannon–Wiener evenness index (E) were below 0.5, indicating that the captured mosquitoes were not evenly distributed throughout the collection method.

**Table-2:** Abundance, richness, diversity, evenness, and dominance of mosquitoes using human landing and resting collection methods in Central Bengkulu Regency, Indonesia, from November 2022 to May 2023.

Indices	Collection method			

	HLC indoor	HLC outdoor	Resting indoor	Resting outdoor
Number of species (S)	14	15	8	11
Number of individuals (N)	105	269	112	322
Dominance (D)	0.302	0.373	0.373	0.445
Simpson (1-D)	0.698	0.6266	0.6269	0.5544
Shannon (H)	1.683	1.279	1.241	1.022
Shannon–Wiener Evennes (E)	0.3843	0.2396	0.4323	0.2526

HLC=Human landing collection

### Density and biting activity of mosquitoes

**Figure-2:**
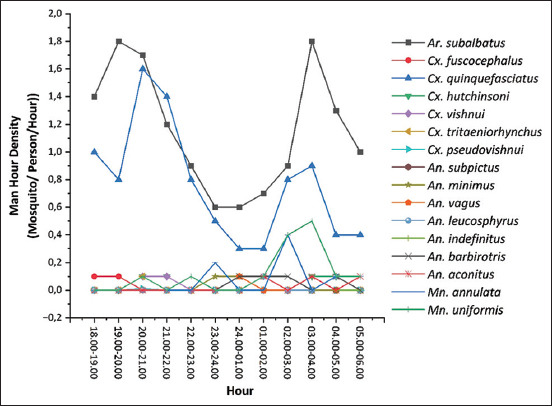
Man-Hour Density Mosquitoes in Central Bengkulu Regency, Indonesia, from November 2022 to May 2023.

Mosquito density and biting activity were calculated using the MBR and MHD. The results of the mosquito density calculation are presented in Table-3, and the results of the mosquito biting activity calculation are presented in Figure-2. The five predominant mosquito species in biting humans were *Ar. subalbatus*, *Cx. quinquefasciatus*, *Cx. pseudovishnui*, *Ma. annulata*, and *Ma. uniformis*, with MBR values of 23.67, 15.33, 3.83, 1.33, and 1.33 mosquitoes per person per night, respectively. The average MBR of mosquitoes in Central Bengkulu Regency from November 2022 to May 2023 was three individuals per night.

**Table-3:** Man-biting rate of mosquitoes and filarial detection in Central Bengkulu Regency, Indonesia, from November 2022 to May 2023.

Species	Density (mosquito/person/night)	Filarial detection	

		ITS-1	HhaI
*Aedes aegypti*	0.17	Negative	Negative
*Armigeres subalbatus*	23.67	Negative	Negative
*Culex fuscocephalus*	0.33	Negative	Negative
*Culex quinquefasciatus*	15.33	Negative	Negative
*Culex hutchinsoni*	0.67	Negative	Negative
*Culex vishnui*	0.33	Negative	Negative
*Culex tritaeniorhynchus*	0.17	Negative	Negative
*Culex pseudovishnui*	3.83	Negative	Negative
*Culex pseudosinensis*	0.00	Negative	Negative
*Anopheles subpictus*	0.50	Negative	Negative
*Anopheles minimus*	0.33	Negative	Negative
*Anopheles vagus*	0.17	Negative	Negative
*Anopheles leucosphyrus*	0.17	Negative	Negative
*Anopheles indefinitus*	0.50	Negative	Negative
*Anopheles barbirotris*	0.17	Negative	Negative
*Anopheles aconitus*	0.17	Negative	Negative
*Mansonia annulata*	1.33	Negative	Negative
*Mansonia uniformis*	1.33	Negative	Negative

ITS-1: Internal transcribed pacer 1, HhaI: *Haemophilus haemolyticus* enzyme I

In this study, mosquitoes fed on humans throughout the night, exhibiting distinct activity patterns. Specifically, *Ar. subalbatus* displayed biting behavior every hour at night, peaking between 7:00 PM and 9:00 PM, with a subsequent increase observed after midnight from 2:00 AM to 4:00 AM. Conversely, *Cx. quinquefasciatus* initiated biting activity from 8:00 PM to 10:00 PM, with the activity tapering off until morning. Similarly, *Ma. annulata* exhibited biting behavior from 2:00 a.m. to 3:00 a.m., which gradually diminished toward dawn, in contrast to the pattern observed in *Ma. uniformis*.

### Diversity of larval species

In total, 485 mosquito larvae were collected, representing eight species across four genera: Three *Culex*, two *Aedes*, two *Anopheles*, and one *Armigeres* species. The distribution of larval species according to habitat type is presented in Table-4, whereas the abundance, richness, diversity, abundance, and dominance of larvae are presented in Table-5.

**Table-4:** Distribution of larval species in Central Bengkulu Regency, Indonesia, from November 2022 to May 2023.

Species	Number of larvae (%)	Habitat type						

		Natural ponds (%)	Artificial ponds (%)	Ditches (%)	Ricefield (%)	Artificial containers (%)	Swamps (%)	Puddles (%)
*Aedes albopictus*	123 (25.4)	7 (9.5)	2 (11.8)	12 (12.4)	14 (18.7)	76 (67.9)	0 (0)	12 (13.8)
*Aedes aegypti*	42 (8.7)	8 (10.8)	1 (5.9)	10 (10.3)	0 (0)	23 (20.5)	0 (0)	0 (0)
*Armigeres subalbatus*	88 (18.1)	43 (58.1)	3 (17.6)	17 (17.5)	12 (16)	2 (1.8)	7 (30.4)	4 (4.6)
*Anopheles vagus*	26 (5.4)	1 (1.4)	2 (11.8)	0 (0)	23 (30.7)	0 (0)	0 (0)	0 (0)
*Anopheles barbirostris*	15 (3.1)	2 (2.7)	0 (0)	0 (0)	12 (16)	0 (0)	1 (4.3)	0 (0)
*Culex pseudovishnui*	20 (4.1)	4 (5.4)	0 (0)	2 (2.1)	2 (2.7)	0 (0)	0 (0)	12 (13.8)
*Culex vishnui*	63 (13.0)	1 (1.4)	2 (11.8)	13 (13.4)	4 (5.3)	6 (5.4)	3 (13)	34 (39.1)
*Culex quinquefasciatus*	108 (22.3)	8 (10.8)	7 (41.2)	43 (44.3)	8 (10.7)	5 (4.5)	12 (52.2)	25 (28.7)
Total	485 (100)	74 (100)	17 (100)	97 (100)	75 (100)	112 (100)	23 (100)	87 (100)

**Table-5:** Abundance, richness, diversity, evenness, and dominance of larvae of mosquito species based on habitats in Central Bengkulu Regency, Indonesia, from November 2022 to May 2023.

Indices	Habitat type						

	Natural ponds	Artificial ponds	Ditches	Ricefield	Artificial container	Swamps	Puddles
Number of species (S)	8	6	6	7	5	4	5
Number of individuals (N)	74	17	97	75	112	23	87
Number of collection points	5	7	19	9	13	23	19
Dominance (D)	0.37	0.25	0.27	0.19	0.51	0.38	0.27
Simpson (1-D)	0.62	0.75	0.73	0.80	0.49	0.62	0.72
Shannon (H)	1.39	1.59	1.51	1.75	0.95	1.10	1.41
Shannon–Wiener Evennes (E)	0.50	0.82	0.75	0.82	0.52	0.75	0.82

Eight larval species were found in natural habitats. *Aedes albopictus* had the highest abundance (25.4%), and it is commonly found in artificial container habitats. *Ar. subalbatus* larvae had an abundance of 18.1% and are frequently found in natural ponds.

The Shannon’s diversity index (H) for diverse habitat types exceeded 1, except for man-made container habitats (0.95). Based on Shannon diversity index (H) values, the diversity of mosquito larvae in Central Bengkulu Regency was generally moderate, except in man-made container habitats. The evenness value of mosquito larvae based on the Shannon–Wiener evenness index (E) approached 1, indicating that larvae tended to be evenly distributed across all habitats.

### Physicochemical and biological habitat characteristics of larvae

Seven habitats were identified as mosquito breeding sites: Natural ponds, artificial ponds, ditches, rice fields, man-made containers, swamps, and puddles. The results of the measurements of the physical and chemical characteristics in the found habitats are presented in Table-6, whereas the results of the measurements of the biological characteristics are presented in Table-7.

**Table-6:** Physicochemical characteristics of larval habitats in Central Bengkulu Regency, Indonesia, from November 2022 to May 2023.

Habitat type	Number of habitats	Average			

		Temperature (°C)	Illumination (Lx)	pH	Salinity (%)
Natural ponds	5 (5.3)	26.2	531	7.2	0
Artificial ponds	7 (7.4)	28.1	634	7.2	0
Ditches	19 (20.0)	25.4	224	7.5	0
Rice field	9 (9.5)	27.3	543	7.1	0
Artificial container	13 (13.7)	28.7	674	7.2	0
Swamps	23 (24.2)	27.6	523	7.5	0
Puddles	19 (20.0)	27.7	497	7.8	0

The most common habitat type (24.2%) was swamps. The average water temperatures in the study habitats ranged from 25.4°C to 28.7°C. Ditches had the lowest water temperature (25.4°C), whereas man-made containers had the highest water temperature (28.7°C). The light intensity varied from 224 to 674 lx, with man-made container habitats having the highest (674 lx) and ditches having the lowest (224 lx). The average pH ranged from 7.1 to 7.9, with rice fields having the lowest pH (7.1) and puddles having the highest pH (7.8). In addition, all larval habitats had a salinity level of zero. More than half of the 95 larval habitats had turbid water (54.7%), vegetation present (58.9%), and no predators (68.4%). Turbid water conditions and vegetation were primarily found in swamp habitats, whereas almost one-third (29.2%) of predator-free habitats were in ditches and puddles.

## Discussion

This study aimed to identify the diversity of mosquito species, analyze their biting activity, identify larval habitat characteristics, and detect the presence of microfilaria to understand the transmission dynamics of filariasis. The epidemiology of filariasis encompasses the complex interactions between microfilariae as a disease agent, humans as a primary host, other host reservoirs, adult mosquitoes as transmission vectors, and the physical and biological environments supporting the transmission cycle [5]. In the context of the Central Bengkulu Regency, this study identified 18 species of mosquitoes belonging to five genera: *Aedes*, *Anopheles*, *Armigeres*, *Culex*, and *Mansonia*. The mosquitoes were classified into the *Culicinae* subfamily, except for *Anopheles*, which belongs to the Anophelinae subfamily. Morphologically, *Aedes* and *Culex* can be distinguished by their wing length and abdomen shape: *Aedes* have a slender abdomen, whereas *Culex* has a narrow abdomen. *Armigeres*, which is morphologically larger than the other members of the *Culicinae* subfamily, has a slightly curved proboscis. *Mansonia* distinguishes itself by its wide, asymmetrical wings and thin abdomen tip [15].

The findings revealed that *Ar. subalbatus* exhibited the highest abundance (44.8%), with a notable tendency toward outdoor biting (49.1%) and resting activities (54.7%). This species has considerable potential as a vector for filariasis, consistent with findings from previous research conducted in Sarmi, Papua, Indonesia [16]. In addition, studies by Intarapuk and Bhumiratama [17] and Siriyasatien *et al*. [18] highlighted the potential of *Ar. subalbatus* as a filariasis vector in Southern Thailand. Moreover, research conducted by Muslim *et al*. [19] in Peninsular Malaysia confirmed the involvement of *Ar. subalbatus* as a zoonotic vector.

In this study, *Cx. quinquefasciatus* exhibited the second-highest indoor biting activity (50.4%) and indoor resting behavior (50.0%). This species acts as a vector for filariasis in humans, as evidenced by findings from previous studies. For instance, Astuti *et al*. [20] estimated its prevalence and role as a filariasis vector in Majalaya District, West Java, Indonesia. Similarly, Ramadhani *et al*. [21] detected stage L3 *W. bancrofti* in *Cx. quinquefasciatus* mosquitoes indoors and outdoors in Pabean Village, Pekalongan, Indonesia, with an infection rate of 34.4%. In addition, *Cx. quinquefasciatus* dominance has been noted in filariasis-endemic regions such as Digha, West Bengal, India [22]. Furthermore, Derua *et al*. [23] reported a 0.23% infection rate of stage L3 *W. bancrofti* in 3,866 *Cx. quinquefasciatus* mosquitoes found on Mafia Island, Tanzania.

The high abundance of *Cx. quinquefasciatus* at the study site had significant implications for public health. As a primary vector for diseases such as LF and the potential transmission of other diseases such as West Nile fever and encephalitis, this mosquito increased the risk of infectious diseases within the community. The impact extended beyond physical aspects, such as chronic swelling, affecting the quality of life and productivity of affected individuals. Therefore, effective vector management and the implementation of appropriate disease prevention strategies are crucial to mitigate the negative health impacts of the abundance of *Cx. quinquefasciatus* in this research area [22].

The diversity of mosquito species in Central Bengkulu Regency was moderate. The predominant species that bite humans include Ar. subalbatus (44.5%), followed by Cx. quinquefasciatus, *Cx. pseudovishnui, Ma. annulata*, and *Ma. uniformis*. These findings indicate an average MBR of three mosquitoes per person per night. Moreover, the biting activity (MHD) exhibited fluctuating patterns throughout the night. Interestingly, the average MBR varied significantly across locations, depending on the environmental context. Supriyono *et al*. [24] reported MBR values ranging from 0.02 to 2.43 bites per person per night in the villages of Hamarung and Hukai in Juai District, Balangan Regency, South Kalimantan Province, with biting activity fluctuating throughout the night. Similarly, Syahrani *et al*. [25] documented an MBR of 10.5 bites per person per night in West Sumba and Southwest Sumba Regencies in East Nusa Tenggara Province, where biting occurred both indoors and outdoors. Consistency in mosquito biting rates, indoors and outdoors underscore the significance of this information for guiding effective vector control strategies.

Furthermore, a study conducted by Ligsay *et al*. [26] demonstrate that the high rate of mosquito vector bites has serious impacts on the community in the research area, with increased disease transmission leading to a significant health burden. These impacts were not only limited to health but also affected the economy because of increased medical costs and reduced productivity [27]. In addition, epidemiological theory has shown that higher densities of mosquito bites increase the risk of disease transmission among the human population [28].

The highest number of mosquito larvae was found in *Ae. Albopictus* (25.4%), followed by *Ar*. *subalbatus* (23%) and *Cx. quinquefasciatus* (22.5%), which were predominantly found in artificial containers. *Ae. albopictus* was predominant in artificial containers (67.9%), *Cx. quinquefasciatus* was found in marshes (52.2%), and *Ar. subalbatus* was commonly found in natural ponds (58.1%). Based on the Shannon–Wiener evenness index, the diversity of mosquito larvae was within the moderate category, whereas an even distribution was observed across all habitat types. The water temperature ranged from 25.4°C to 28.7°C, with an average illumination of 224–674 lx and pH levels ranging from 7.1 to 7.9. The habitat characteristics supportive of breeding included murky water conditions (54.7%), the presence of vegetation (58.9%), and the absence of predators (68.4%) (Table-7). Geographically, the research location is surrounded by rubber plantations, oil palm plantations, and agricultural fields, with the majority of the population engaged in rubber and oil palm farming and conducting activities in the fields from night to day. These activities facilitate contact between vector mosquitoes and humans. Arsin [29] reported that mosquito abundance is influenced by physical and chemical factors in breeding grounds, with optimal temperatures ranging from 25°C to 27°C. In contrast, extreme temperatures can hinder larval growth or kill larvae. Rajeswari and Nagarajan [30] reported that mosquito diversity and abundance are influenced by the availability of breeding sites, resting places, and climate, particularly temperature and rainfall. If available breeding sites are more suitable, the mosquito population will become denser, increasing the likelihood of disease transmission to humans [31].

**Table-7:** Biological characteristics of larval habitats in Central Bengkulu Regency, Indonesia, from November 2022 to May 2023.

Habitat type	Number of habitat (%)	Turbidity		Presence of vegetation		Presence of the predator	
		
		No turbidity (%)	Turbid (%)	Presence (%)	Absent (%)	Presence (%)	Absent (%)
Natural ponds	5 (5.3)	1 (2.3)	4 (7.6)	5 (8.9)	0 (0.0)	5 (16.6)	0 (0.0)
Artificial ponds	7 (7.4)	5 (11.6)	2 (3.8)	5 (8.9)	2 (5.1)	1 (3.3)	6 (9.2)
Ditches	19 (20.0)	11 (25.6)	8 (15.3)	2 (3.6)	17 (43.6)	0 (0.0)	19 (29.2)
Rice field	9 (9.5)	2 (4.6)	7 (13.4)	6 (10.7)	3 (7.7)	7 (23.3)	2 (3.1)
Artificial container	13 (13.7)	12 (27.9)	1 (1.9)	0 (0.0)	13 (33.3)	0 (0.0)	13 (20.0)
Swamps	23 (24.2)	1 (2.3)	22 (42.3)	23 (41.1)	0 (0.0)	17 (56.6)	6 (9.2)
Puddles	19 (20.0)	11 (25.6)	8 (15.3)	15 (26.8)	4 (10.3)	0 (0.0)	19 (29.2)
Total	95 (100)	43 (45.2)	52 (54.7)	56 (58.9)	39 (41.0)	30 (31.5)	65 (68.4)

The PCR of the collected mosquitoes did not detect L3 filarial worms. PCR can detect L3 filarial worms in small quantities. However, this method is subject to limitations, such as potential DNA degradation due to repeated freeze-thaw cycles and improper DNA isolation storage techniques, as noted by Tan *et al*. [32]. Similarly, Nirwan *et al*. [33] reported the absence of L3 filarial worms in mosquitoes captured in Bogor Regency, Central Java, Indonesia, which was likely due to factors such as inadequate sample size, limited study duration, and the possibility of false-negative results arising from PCR examination using pooled mosquito samples. Despite the absence of L3 filarial worms in the mosquitoes tested, this does not necessarily indicate the absence of filariasis transmission in Central Bengkulu Regency, Bengkulu Province. Continuous early warning against filariasis transmission is extremely important, particularly considering the latest data in 2020, which identified new cases of filariasis at our research site, indicating the existence of sources of filariasis transmission [34]. The dominant mosquito species captured at the study site were previously identified as primary filariasis vectors in various regions across Indonesia.

## Conclusion

This study identified 18 mosquito species from five genera in Central Bengkulu Regency, including *Ar. subalbatus*, which is a potential vector for filariasis with significant outdoor biting and resting activity. *Cx. quinquefasciatus* also emerged as an important indoor vector for filariasis, with serious implications for public health. The larval habitats were mostly artificial containers for *Ae. albopictus*, swamps for *Cx. quinquefasciatus*, and natural ponds for *Ar. subalbatus*. Although PCR analysis did not detect L3 filarial worms in the mosquitoes collected, the presence of this main vector for filariasis is an important consideration for continuous vector surveillance and transmission in Central Bengkulu Regency, Indonesia. Further research is needed to understand the role of other reservoir hosts and the interactions between various mosquito species in supporting filariasis transmission.

## Authors’ Contributions

DK: Designed the study, conducted field surveys, and collected mosquito samples. DK and SU: Performed the laboratory examination. UKH, SS, and RT: Designed the study, performed the laboratory examination and drafted and revised the manuscript. All authors have read, reviewed, and approved the final manuscript.
